# Catalytically important damage-free structures of a copper nitrite reductase obtained by femtosecond X-ray laser and room-temperature neutron crystallography

**DOI:** 10.1107/S2052252519008285

**Published:** 2019-06-23

**Authors:** Thomas P. Halsted, Keitaro Yamashita, Chai C. Gopalasingam, Rajesh T. Shenoy, Kunio Hirata, Hideo Ago, Go Ueno, Matthew P. Blakeley, Robert R. Eady, Svetlana V. Antonyuk, Masaki Yamamoto, S. Samar Hasnain

**Affiliations:** a Molecular Biophysics Group, Institute of Integrative Biology, Faculty of Health and Life Sciences, University of Liverpool, Liverpool L69 7ZB, England; b SR Life Science Instrumentation Unit, RIKEN SPring-8 Centre, Sayo 679-5148, Japan; c Large-Scale Structures Group, Institut Laue–Langevin, 71 Avenue des Martyrs, 38000 Grenoble, France

**Keywords:** copper-containing nitrite reductases, neutron crystallography, X-ray free-electron lasers

## Abstract

Copper-containing nitrite reductases (CuNiRs) that convert NO_2_^−^ to NO via a Cu_CAT_–His–Cys–Cu_ET_ proton-coupled redox system are of central importance in nitrogen-based energy metabolism. These metalloenzymes, like all redox enzymes, are very susceptible to radiation damage from the intense synchrotron-radiation X-rays that are used to obtain structures at high resolution. Understanding the chemistry that underpins the enzyme mechanisms in these systems requires resolutions of better than 2 Å. Here, for the first time, the damage-free structure of the resting state of one of the most studied CuNiRs was obtained by combining X-ray free-electron laser (XFEL) and neutron crystallography. This represents the first direct comparison of neutron and XFEL structural data for any protein. In addition, damage-free structures of the reduced and nitrite-bound forms have been obtained to high resolution from cryogenically maintained crystals by XFEL crystallography. It is demonstrated that Asp_CAT_ and His_CAT_ are deprotonated in the resting state of CuNiRs at pH values close to the optimum for activity. A bridging neutral water (D_2_O) is positioned with one deuteron directed towards Asp_CAT_ O^δ1^ and one towards His_CAT_ N^∊2^. The catalytic T2Cu-ligated water (W1) can clearly be modelled as a neutral D_2_O molecule as opposed to D_3_O^+^ or OD^−^, which have previously been suggested as possible alternatives. The bridging water restricts the movement of the unprotonated Asp_CAT_ and is too distant to form a hydrogen bond to the O atom of the bound nitrite that interacts with Asp_CAT_. Upon the binding of NO_2_^−^ a proton is transferred from the bridging water to the O^δ2^ atom of Asp_CAT_, prompting electron transfer from T1Cu to T2Cu and reducing the catalytic redox centre. This triggers the transfer of a proton from Asp_CAT_ to the bound nitrite, enabling the reaction to proceed.

## Abstract 2

Damage-free structures of the resting state of one of the most studied copper nitrite reductases were obtained independently by X-ray free-electron laser (XFEL) and neutron crystallography, representing the first direct comparison of neutron and XFEL structural data for any protein. In addition, damage-free structures of the reduced and nitrite-bound forms have been obtained to high resolution from cryogenically maintained crystals by XFEL crystallography.

## 1. Introduction   

The highly brilliant undulator beamlines at modern synchrotron facilities have facilitated the structure determination of biological molecules and their complexes at high resolution using conventional synchrotron-radiation crystallography (SRX). The brilliance of the X-rays at some of the state-of-the-art crystallographic beamlines has enabled this to be achieved using much smaller (10–30 µm) crystals than was anticipated at the turn of the century. These gains have come at the expense of an increased absorbed X-ray dose per unit volume and the potential for concomitant radiolysis and radiation damage (Garman, 2010 ▸; Yano *et al.*, 2005 ▸; Horrell *et al.*, 2016 ▸). Biological molecules and their complexes that use the redox properties of transition metals are particularly sensitive to radiation damage and X-ray-induced chemical changes (Yano *et al.*, 2005 ▸; Horrell *et al.*, 2016 ▸). This poses a serious limitation to obtaining damage-free structures of redox enzymes at sufficiently high resolutions that the enzyme mechanism can be defined at the chemical level, such as changes resulting from one-electron reduction or the geometry and position of the chemical substrate. The recent advent of XFEL crystallography using femtosecond X-ray pulses provides a new opportunity to obtain damage-free structures (Suga *et al.*, 2014 ▸, 2017 ▸), adding to neutron crystallography and NMR, which have remained the only radiation-damage-free structural probes for decades, albeit with their own particular limitations (Blakeley *et al.*, 2015 ▸; Blakeley, 2009 ▸; Luchinat & Banci, 2017 ▸).

Copper nitrite reductases (CuNiRs) are a highly conserved family of enzymes involved in the first committed step in the denitrification pathway: the reduction of nitrite (NO_2_^−^) in a one-electron two-proton reaction to form the gaseous product nitric oxide (NO; Zumft, 1997 ▸). The enzymes are homotrimeric, with two cupredoxin-like domains in each monomer that harbour one type 1 copper (T1Cu) and one type 2 copper (T2Cu) site. The catalytic T2Cu site is located at the interface between two monomers, with a (His)_3_–H_2_O ligand coordination, while the T1Cu site is located near the surface of the protein. The two copper ions are hard-wired *via* a 12.1 Å Cys–His bridge through which an electron required for catalysis is transferred when the substrate NO_2_^−^ binds to the oxidized T2Cu, displacing the bound water ligand of the resting enzyme. NO_2_^−^ accesses the active site via a hydrophobic channel of ∼6 Å in width that is formed at the monomer–monomer boundary. Two residues, Asp98 (Asp_CAT_) and His255 (His_CAT_) in *Achromobacter cycloclastes* CuNiR (*Ac*NiR) numbering, that are conserved in all CuNiRs and are located either side of the T2Cu, are required for enzymatic activity. Asp_CAT_ is hydrogen-bonded to the copper H_2_O ligand and also to His_CAT_ via an H_2_O bridge. This hydrogen-bonding network is preserved when nitrite binds, and mutagenic, mechanistic and computational studies are consistent with its involvement in the provision of the two protons for the reaction. However, an unresolved issue in mechanistic studies is where the two protons that are required for catalysis originate from. Protonated nitrite is an unlikely source since when bound to Cu^2+^ it is energetically unstable, decaying to the damaging NO^+^. In addition to its role in proton delivery, Asp_CAT_ is involved in modulating nitrite binding. In *Ac*NiR the Asp_CAT_ residue has been observed in two different conformations: one termed ‘proximal’, in which it is oriented towards the T2Cu and linked to His255 via a water molecule, and a second position termed ‘gatekeeper’, in which it is oriented away from the T2Cu and hydrogen-bonded to the H_2_O ligand of the T2Cu (Antonyuk *et al.*, 2005 ▸). Synchrotron-based X-ray structural studies have shown that nitrite binds to the oxidized T2Cu site of CuNiR to displace the H_2_O ligand. In most structures, the binding mode is a bidentate η^2^-O,O binding (Solomon *et al.*, 2014 ▸), with a single example of η^1^-O binding observed in an enzyme from a thermophile (Fukuda *et al.*, 2014 ▸). A number of different orientations of η^2^-O,O-bound nitrite have been observed, both in the resting enzyme, which shift with pH, and also in X-ray-induced turnover. In the case of *Ac*NiR the serial structures used to construct a structural movie, which were obtained by low-dose MSOX (multiple serial structures from one crystal) at 190 K, revealed a ‘top-hat’ (vertical O-bidentate) conformation of NO_2_^−^ in the first frame of structures, which then flipped to a ‘side-on’ conformation with near-equivalent Cu—O1, Cu—O2 and Cu—N distances in subsequent frames, prior to bond breakage and product formation (Horrell *et al.*, 2018 ▸). Although this may indicate that the first stage of substrate utilization may involve a conformational change, pH-dependence of the NO_2_^−^ binding geometry has been observed for some CuNiRs from other species (Zhao *et al.*, 2002 ▸; Abraham *et al.*, 1997 ▸; Jacobson *et al.*, 2007 ▸).

Significant efforts have been made using XFEL crystallo­graphy to obtain damage-free structures of the resting state as well as a number of catalytically important forms for a number of CuNiRs [NiRs from *Alcaligenes faecalis* (*Af*NiR; Fukuda, Tse, Nakane *et al.*, 2016 ▸), *Geobacillus thermodenitrificans* (*Gt*NiR; Fukuda, Tse, Suzuki *et al.*, 2016 ▸) and *Alcaligenes xylosoxidans* (*Ax*NiR; Halsted *et al.*, 2018 ▸)]. In the resting-state serial femtosecond crystallography (SFX) structures of *Af*NiR and *Gt*NiR, a chloride and a sodium ion originating from the crystallization or purification protocols were found in the T2Cu pocket, respectively. For *Ax*NiR, serial femtosecond rotational crystallography (SF-ROX) revealed an unprecedented dioxo ligand bound to the T2Cu site, which was interpreted to rationalize the oxidase activity of some CuNiRs and was considered to represent a trapped intermediate of the oxidase reaction awaiting the delivery of a second electron to enable turnover to produce the product H_2_O_2_ (Halsted *et al.*, 2018 ▸).

Here, using SF-ROX, damage-free structures of *Ac*NiR have been obtained in the as-isolated oxidized, chemically reduced and NO_2_^−^-bound forms. These are complemented by a damage-free room-temperature (RT) neutron crystallographic structure of the as-isolated perdeuterated enzyme. As such, unprecedented information is obtained on the nature of the ligands of T2Cu in the resting state and the protonation states of the catalytic residues. Neutron diffraction studies provide the positions of D atoms, allowing the direct determination of the protonation states of the protein residues and water molecules in the catalytic pocket for the first time, both of which are necessary in order to understand the enzyme mechanism. We unequivocally establish that in the resting state the T2Cu of *Ac*NiR is liganded by a single, neutral water molecule. Moreover, the neutron structure shows Asp_CAT_ to be unprotonated, which is consistent with the consensus view (Solomon *et al.*, 2014 ▸), but, contrary to expectation, shows that His_CAT_ is also deprotonated at the N^∊2^ position. These observations, together with damage-free SF-ROX structures of the reduced and substrate-bound forms, provide new insights into the catalytic steps of these important enzymes. These structures are also likely to provide a firm basis for detailed computational chemistry efforts.

## 2. Methods   

### 2.1. Production of recombinant *Ac*NiR crystals   

The *nirK* gene from *A. cycloclastes* with codon optimization for expression in *Escherichia coli* was acquired from GenScript and cloned into a pET-26b(+) plasmid. The plasmid was transformed into *E. coli* BL21 (DE3) cells via heat shock and the transformant was cultured on Kan^R^ lysogeny broth (LB) agar to isolate individual colonies. 500 ml LB supplemented with 30 µg ml^−1^ kanamycin was inoculated with a single colony and was incubated with shaking at 37°C. Protein overexpression was induced with 2 m*M* isopropyl β-d-1-thiogalactopyranoside (IPTG) and 1 m*M* CuSO_4_. Incubation continued for 24 h, after which the cells were harvested by centrifugation and resuspended in 20 m*M* Tris–HCl pH 7.5, 0.1 mg ml^−1^ lysozyme before being disrupted by sonication. The lysate was collected by centrifugation and dialyzed against 20 m*M* Tris–HCl pH 7.5, 2 m*M* CuSO_4_, followed by dialysis against water. The lysate was loaded onto a DEAE-Cellulose column equilibrated with 20 m*M* Tris–HCl pH 7.5, which was subsequently washed with 20 m*M* Tris–HCl pH 7.5 followed by 100 m*M* Tris–HCl pH 7.5. *Ac*NiR was eluted from the column using an NaCl gradient from 100 to 250 m*M* in 20 m*M* Tris–HCl pH 7.5. 4 *M* ammonium sulfate was used to completely precipitate the *Ac*NiR, which was dissolved in 10 m*M* HEPES–NaOH pH 6.5. The *Ac*NiR was concentrated to 50 mg ml^−1^ and was crystallized by hanging-drop vapour diffusion against a 1:1 ratio of 1.2 *M* ammonium sulfate and 100 m*M* citrate buffer pH 5.0. Crystals with a pyramidal shape grew to ∼0.7 × 0.7 × 0.7 mm in size.

### 2.2. SF-ROX crystal treatment, data collection and processing   

The harvested crystals of *Ac*NiR were soaked in cryoprotectant, cryocooled by plunging into liquid nitrogen and maintained at 77 K after cooling. SF-ROX^OX^ crystals were soaked in 3.4 *M* ammonium sulfate, 100 m*M* citrate buffer pH 5.0 for 10 s. SF-ROX^NIT^ crystals were soaked in 3.4 *M* sodium malonate pH 5.0, 100 m*M* sodium nitrite for 10 s. SF-ROX^RED^ crystals were soaked in 3.4 *M* sodium malonate pH 5.0, 100 m*M* sodium ascorbate for 30 min, after which their colour changed from green to colourless. SF-ROX data collection was carried out on BL2 EH3/4b at SACLA at 100 K as described previously (Hirata *et al.*, 2014 ▸; Halsted *et al.*, 2018 ▸). The X-ray energy was set to 10 keV and the pulses were of <10 fs in duration. The sample was positioned 10 mm downstream of the XFEL focal point, which gave a beam size at the sample position of 2.2 × 4.5 µm. The XFEL beam was atten­uated to 5.7 × 10^10^ photons per pulse using a 100 µm thick aluminium X-ray attenuator. The crystals were rotated 0.1° and translated 50 µm between each snapshot. X-ray diffraction images were collected on an MX225-HS CCD detector (Rayonix) with a camera length of 110 mm. The same data-collection and processing procedure was used for all three data sets. Hit finding, indexing and integration were performed using *CrystFEL* (v.0.6.3; White *et al.*, 2016 ▸), with the inner, middle and outer integration radii set to four, five and seven pixels, respectively. After the resolution of the indexing ambiguity, Bragg intensities were merged using the Monte Carlo method with frame scaling. Refinement was carried out in *REFMAC*5 (Murshudov *et al.*, 2011 ▸) using the resting-state *Ac*NiR structure (PDB entry 2bw4; Antonyuk *et al.*, 2005 ▸) as the starting model with riding H atoms and isotropic *B* factors. *Coot* (Emsley *et al.*, 2010 ▸) was used for manual model building between rounds of refinement. Double conformations of the side chains were assigned where 2*F*_o_ − *F*_c_ electron-density maps showed them clearly. The occupancies of the different conformers were chosen by examining the levels of the OMIT (*F*_o_ − *F*_c_) electron-density maps and refined *B* factors. The final quality of the models was assessed using *MolProbity* (Chen *et al.*, 2010 ▸). Data-processing and refinement statistics are given in Table 1 ▸.

### 2.3. In-house laboratory-source data collection and processing   

Data sets for the resting state at a variety of pH values were obtained at the Barkla X-ray Laboratory of Biophysics using a Rigaku FR-E+ SuperBright rotating-anode generator with an EIGER R 4M detector. The same experimental setup was used to obtain a room-temperature structure from a perdeuterated crystal. For room-temperature data collection, the crystal was mounted in the cryoloop and protected from drying using MicroRT Tubing (MiTeGen) containing reservoir solution. Crystals of *Ac*NiR were soaked for 15 min in 3.1 *M* sodium malonate, 100 m*M* NaNO_2_ at pH 5.0, 5.5, 6.0 and 6.5. For each pH value, data sets were collected with 15 s exposure time and a 1.0° oscillation step per image, with a total of 60 images per data set. The images were processed using *XDS* (Kabsch, 2010 ▸) and *AIMLESS* (Evans & Murshudov, 2013 ▸), and refinement, model building and validation were carried out in the same manner as for the SF-ROX structures. Data-processing and refinement statistics can be found in Supplementary Table S1.

### 2.4. Production of perdeuterated *Ac*NiR crystals   

An *E. coli* cell pellet containing *Ac*NiR expressed under perdeuterated conditions was produced by the Deuteration Laboratory at the Institut Laue–Langevin (Haertlein *et al.*, 2016 ▸). 5 g of the pellet was resuspended in lysis buffer consisting of 20 m*M* Tris–HCl pH 7.5, 150 m*M* NaCl, 0.1 mg ml^−1^ lysozyme, 1 µg ml^−1^ protease inhibitors, 0.1 µg ml^−1^ DNAse and stirred on ice for 30 min. The cells were subsequently disrupted by sonication and the lysate was collected by centrifugation. The cell lysate was dialyzed against 20 m*M* Tris–HCl pH 7.5, 1 m*M* CuSO_4_ followed by the addition of 2 µl H_2_O_2_ and further dialysis against the same buffer without any additives. The lysate was loaded onto a hydroxyapatite column equilibrated with 100 m*M* Tris–HCl pH 7.5 and washed with a mixture of 6 m*M* Tris–HCl pH 7.5 and 2 m*M* potassium phosphate buffer pH 7.5 before being eluted using a potassium phosphate buffer gradient from 10 to 150 m*M*. The eluted fraction was applied onto a DEAE-Sepharose column pre-equilibrated with 20 m*M* Tris–HCl pH 7.5 and was eluted using an NaCl gradient from 50 to 250 m*M*. The eluted fraction was subsequently applied onto a HiLoad 16/600 Superdex 200 size-exclusion chromatography column (GE Life Sciences) pre-equilibrated with 150 m*M* NaCl in 20 m*M* Tris–HCl pH 7.5. In the final stage, *Ac*NiR was precipitated using 4 *M* ammonium sulfate and the pellet was resuspended in 50 m*M* MES–NaOH pD 6.9. The pH values were determined using a conventional pH meter and the pH_obs_ reading was corrected as described in Schowen & Schowen (1982 ▸). *Ac*NiR was crystallized by hanging-drop vapour diffusion against a 1:1 ratio of 1.1 *M* ammonium sulfate and 100 m*M* sodium acetate pD 5.4. For crystallization, 5 µl protein solution at a concentration of 20 mg ml^−1^ was mixed with 5 µl reservoir solution; crystallization was initiated by adding microcrystals 2 h after the crystallization was set up. Two additional drops of equivalent size were added in one week and were merged with the nucleated drop after equilibration. It took three weeks for the crystal to reach its final size. A large single pyramid-shaped crystal of ∼0.9 × 0.4 × 1.0 mm was mounted in a 2 mm diameter capillary and stored for neutron data collection.

### 2.5. Neutron data collection and structural refinement   

Neutron diffraction data were collected at RT to 1.8 Å resolution from a perdeuterated crystal of *Ac*NiR (∼0.36 mm^3^ in volume) using the quasi-Laue neutron diffractometer LADI-III (Blakeley *et al.*, 2010 ▸) at the Institut Laue–Langevin. A total of 20 images of 18 h exposure time each were collected from four different crystal orientations. These data were indexed and integrated using *LAUEGEN* (Campbell *et al.*, 1998 ▸), wavelength-normalized using *LSCALE* (Arzt *et al.*, 1999 ▸) and scaled and merged using the *CCP*4 program *SCALA* (Winn *et al.*, 2011 ▸). Previously, D-exchanged crystals of similar volume had been used to collect diffraction data that extended to only 2.3 Å resolution (Blakeley *et al.*, 2015 ▸), illustrating the benefits of using perdeuterated samples.

A 1.9 Å resolution X-ray data set collected at RT from a perdeuterated crystal was used as the starting model for neutron structural refinement. Using *Ready_Set!* from the *PHENIX* software suite (Adams *et al.*, 2010 ▸), D atoms were added to the residues at calculated positions in preparation for structural refinement using *phenix.refine*. After initial rigid-body refinement, several rounds of maximum-likelihood-based refinement of individual coordinates and individual *B* factors against the neutron data were performed while applying restraints from the X-ray structure of the perdeuterated crystal, the data for which were collected at room temperature using an in-house X-ray generator, to maintain the geometry of the copper sites. After every round the model was visually inspected and manipulated in *Coot* (Emsley & Cowtan, 2010 ▸) using both positive and negative *F*_o_ − *F*_c_ and 2*F*_o_ − *F*_c_ nuclear scattering-length density maps to guide the modelling of solvent and protein D atoms. The final model contained 179 water molecules that were observed as full D_2_O molecules, along with ten water molecules that were rotationally disordered and thus were included as O atoms only. Data-processing and refinement statistics can be found in Table 2 ▸.

## 3. Results   

### 3.1. Resting-state structures of *Ac*NiR determined by SF-ROX and neutron crystallography   

**Figure 1:**
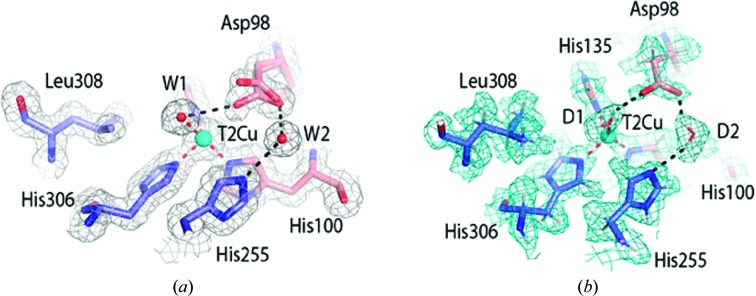
The T2Cu site of *Ac*NiR determined by SF-ROX and neutron crystallography. (*a*) The T2Cu in SF-ROX^OX^ is ligated by a single water molecule (W1) hydrogen-bonded to the Asp98 residue. Asp98 (Asp_CAT_) is visible in two conformations in the proximal position, with the residue rotating around the fixed O^δ1^ atom. Asp_CAT_ is subsequently hydrogen-bonded to the linking water (W2), which is hydrogen-bonded to His255. Water molecules are shown as red spheres. (*b*) The T2Cu catalytic site determined by neutron crystallography. The protonation states of the T2Cu site residues are clearly seen, along with the orientations of the catalytic D_2_O molecule (D1) and the proton-sharing D_2_O molecule D2. There is no expected protonation of His255 and Asp98, while a D2 molecule connects His255 and Asp98. The 2*F*_o_ − *F*_c_ electron-density map is contoured at the 1σ level and is shown as a grey mesh. The 2*F*_o_ − *F*_c_ nuclear scattering-length density map is contoured at the 1σ level and is shown as a cyan mesh. Atoms are coloured by element, with different colour schemes used for the different chains. The T2Cu is shown as a cyan sphere and D_2_O water molecules are shown as red and white sticks. Metal-coordinating bonds are shown as red dotted lines. Selected hydrogen bonds are shown as black dotted lines.

**Figure 2:**
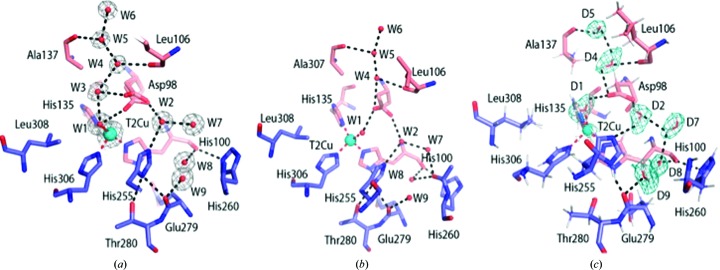
Water structure in the catalytic pocket and substrate-entry channel. (*a*) In the structure of oxidized *Ac*NiR determined by SF-ROX, Asp98 (Asp_CAT_) has a dual conformation; the usual proximal conformation hydrogen-bonds to two waters (W3 and W4), while the distorted proximal position, which is observed for the first time, hydrogen-bonds directly to the T2Cu water ligand. The waters (W3 and W4) are part of the ordered water network in the substrate-entry channel. Both proximal conformations of Asp_CAT_ are hydrogen-bonded via O^δ1^, with the water W2 linking His255 (His_CAT_) to Asp_CAT_. 2*F*_o_ − *F*_c_ electron density is contoured at the 1σ level and is shown as a grey mesh. (*b*) In the atomic resolution crystal structure (PDB entry 2bw4) the proximal conformation is hydrogen-bonded to the ligated water W1A with an occupancy of 0.8. Water W1B with an occupancy of 0.2 is not shown for simplicity. (*c*) In the neutron^OX^ structure, Asp_CAT_ is in a single proximal conformation. The 2*F*_o_ − *F*_c_ nuclear scattering-length density map is contoured around selected heavy waters at the 1σ level and is shown as a teal mesh. Atoms are coloured by element, with different colour schemes used for the different chains. The T2Cu is shown as a cyan sphere, D_2_O water molecules are shown as red and white sticks and water molecules are shown as small red spheres. Metal-coordinating bonds are shown as red dotted lines. Selected hydrogen bonds are shown as black dotted lines.

Resting-state structures of *Ac*NiR were obtained using both SF-ROX at 100 K and neutron crystallography at RT and were refined to 1.5 and 1.8 Å resolution, respectively (Tables 1 ▸ and 2 ▸). The SF-ROX^OX^ structure was compared with the 0.90 Å resolution synchrotron-radiation (SR) structure of *Ac*NiR (Antonyuk *et al.*, 2005 ▸), revealing conservation of the overall structure with an all-protein-atom r.m.s.d. of 0.29 Å. The T2Cu is ligated by a single, highly ordered water molecule (W1) bound in a distorted tetrahedral geometry relative to the histidine plane. The active-pocket residues His_CAT_ and Ile257 (Ile_CAT_) were in similar positions; however, there were marked differences in the positioning of the Asp_CAT_ residue and its hydrogen-bonding network. In the SF-ROX^OX^ structure the proximal conformation of the Asp_CAT_ side chain has two variants of the proximal conformation compared with a single proximal conformation in the synchrotron structure (Figs. 1 ▸ and 2 ▸). The additional conformation is formed by a 34° rotation around the O^δ1^ atom, with the carboxyl O^δ2^ atom forming a hydrogen bond to W1 at 3 Å, while the O^δ2^ atom of the original conformation makes two hydrogen bonds to two water molecules, W3 and W4, one of which, W3, subsequently hydrogen-bonds to the T2Cu-ligated W1. These water molecules are part of the ordered water network in the substrate-entry channel. Both conformations are hydrogen-bonded via the O^δ1^ atom to water W2, linking His255 to Asp98 (Fig. 2 ▸).

The neutron structure was modelled using the 1.9 Å resolution room-temperature X-ray structure of the perdeuterated protein obtained in this study. Subsequently, the structure was refined against the neutron data only, with restraints from the starting model to compensate for the weaker nuclear scattering length from Cu and S atoms. In contrast, the deuterons of the deuterated enzyme and the water molecules have neutron scattering lengths that are similar to those of C atoms. This makes the deuterons of histidine and water, for example, very visible in the neutron map. A water molecule appears as three atoms with similar densities. In contrast, H/D atoms are essentially invisible at the typical resolutions of X-ray structures. Even the subatomic resolution structure of *Ac*NiR at better than 0.9 Å resolution was unable to provide the positions of many of the key H atoms in the catalytic pocket (Blakeley *et al.*, 2015 ▸). Furthermore, the information in these very high-resolution SR structures is compromised as a significantly high X-ray dose is required that results in changes from the dose-dependent solvated electrons. The neutron structure determined here to 1.8 Å resolution provides the location of deuterons in the catalytic core and its associated water network for the first time (Figs. 1 ▸ and 2 ▸). The T2Cu is coordinated by a neutral D_2_O molecule similar to W1 in the distorted tetrahedral position observed in the SF-ROX^OX^ structure.

In the atomic resolution SR structure the T2Cu has a tetrahedral coordination, with W1 hydrogen-bonded to the O^δ2^ atom of the proximal Asp_CAT_ at a distance of 2.8 Å. The position of W1 in the SR structure differs from that in damage-free structures (Fig. 2 ▸). The neutron structure clearly shows W1 to be a D_2_O molecule rather than a D_3_O^+^ or OD^−^ ion, which have previously been suggested as possible alternatives. The Asp_CAT_ residue in the neutron^OX^ structure adopts a single proximal conformation and is bonded to a neutral heavy water D_2_O, which is also hydrogen-bonded to His_CAT_. Compared with the SF-ROX^OX^ structure, the His255 plane undergoes a 20° rotation. The neutron data revealed an ordered network of heavy water molecules around His_CAT_, and also revealed hydrogen-bonding of the deuterated His255 N^∊1^ atom to the carbonyl O atom of Glu279 only [Fig. 2 ▸(*b*)]. An unresolved question in mechanistic studies of CuNiRs is the origin of the protons that are required for the reduction of NO_2_^−^. Several studies involving intramolecular electron-transfer rates and pH-dependent activity, together with computational studies, have suggested the involvement of protonated Asp_CAT_ and His_CAT_ in providing the two protons during catalysis (Ghosh *et al.*, 2009 ▸). Our neutron structure provides unequivocal data on the protonation states of active-site residues in the resting state of the CuNiR enzymes for the first time. The nuclear density maps clearly reveal that neither of these residues are protonated at pD 5.4, where the activity of the enzyme is at a maximum, while the bridging D_2_O has its two O—D bonds directed towards Asp_CAT_ and His_CAT_. There is a chain of fully deuterated waters within hydrogen-bonding distance of each other, close to the liganded water at the T2Cu (Fig. 2 ▸).

The T1Cu site in the neutron structure shows no change in its copper geometry compared with the SF-ROX structure, but the second-sphere amino acid Met141 adopts a single conformation in the neutron^OX^ structure as opposed to a dual conformation in the SF-ROX^OX^ structure. Most of the differences in backbone structural alignment are found in an area of surface loop adjacent to Met141 consisting of residues 187–206, with an all-protein-atom r.m.s.d. of 1.02 Å (Supplementary Fig. S1). The loop is fully occupied and ordered in the neutron structure compared with the partially disordered loop in the SF-ROX^OX^ structure. This loop is associated with the binding of the cognate partner protein cytochrome *c*_551_ (Nojiri *et al.*, 2009 ▸).

### 3.2. SF-ROX structures of the NO_2_^−^-bound form of *Ac*NiR   

**Figure 3:**
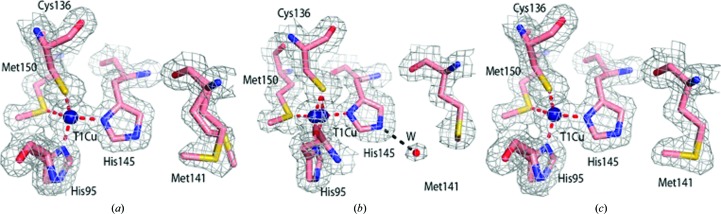
The damage-free T1Cu site in the SF-ROX structures of *Ac*NiR in (*a*) oxidized, (*b*) reduced and (*c*) nitrite-bound forms. 2*F*_o_ − *F*_c_ electron density is contoured at the 1σ level and is shown as a grey mesh. Atoms are coloured by element. The T1Cu is shown as a dark blue sphere and water molecules are shown as small red spheres. Metal-coordinating bonds are shown as red dotted lines. Selected hydrogen bonds are shown as black dotted lines.

**Figure 4:**
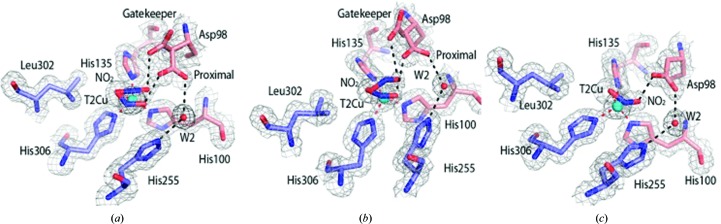
NO_2_^−^-bound T2Cu site of *Ac*NiR. (*a*) NO_2_^−^ is bound to the T2Cu in two conformations in the SF-ROX^NIT^ structure: top-hat and side-on conformations with equal occupancy (50% each). Asp98 is visible in both proximal and gatekeeper conformations, with the gatekeeper conformation corresponding to the top-hat NO_2_^−^ in the SF-ROX^NIT^ structure. (*b*) Conformation of nitrite at pH 5.0 obtained using a low-dose home source. (*c*) At pH 6.5 only a single side-on conformation is visible corresponding to a single Asp98 (Asp_CAT_) proximal position. The half-occupancy water molecule is also bound to T2Cu in the same conformation as in the SF-ROX^OX^ structure. 2*F*_o_ − *F*_c_ electron density is contoured at the 1σ level and is shown as a grey mesh. Atoms are coloured by element, with different colour schemes used for the different chains. The T2Cu is shown as a cyan sphere and water molecules are shown as small red spheres. Metal-coordinating bonds are shown as red dotted lines. Selected hydrogen bonds are shown as black dotted lines.

Upon NO_2_^−^ soaking of crystals of the oxidized enzyme, no changes in the geometry of the T1Cu site were observed in the SF-ROX^NIT^ structure determined at 1.5 Å resolution (Table 1 ▸). Met141 is stabilized in a single conformation, covering His145 [Figs. 3 ▸(*a*) and 3 ▸(*c*)]. A large patch of positive electron density was observed at the T2Cu site, and NO_2_^−^ was initially assigned with full occupancy with a ‘side-on’ binding mode in view of the recent MSOX results (Horrell *et al.*, 2018 ▸). This, however, did not fully satisfy the electron density, and the density was finally assigned as NO_2_^−^ bound in both ‘side-on’ and ‘top-hat’ conformations in almost equal proportions (Supplementary Fig. S2). The O_1_ atoms of ‘top-hat’ and ‘side-on’ NO_2_^−^ are separated by 1.3 Å. A partial-occupancy water (W4) is present at the position of the proximal Asp98 O^δ1^ when in the gatekeeper conformation and is hydrogen-bonded to the bridging water W2. The observation of both conformations of nitrite in the damage-free SF-ROX structure raises an important question regarding the origin of the conformational changes observed during enzyme turnover in the initial frames of MSOX structures. Consistent with the occupancy of the two conformations observed in SF-ROX^NIT^, Asp_CAT_ adopts the proximal and gatekeeper conformations with equal occupancy [Fig. 4 ▸(*a*)]. Based on the possibility of steric interaction, the proximal Asp_CAT_ conformation coincides with ‘side-on’ NO_2_^−^, while the gatekeeper conformation matches the ‘top-hat’ mode. The distorted proximal conformation seen in the SF-ROX^OX^ structure is not visible here. In the atomic resolution SR structure of nitrite-bound *Ac*NiR (PDB entry 2bwi; Antonyuk *et al.*, 2005 ▸), where significant radiolysis would be expected to have occurred, the NO_2_^−^ ion takes up an intermediate position between the dual conformations observed here in the SF-ROX structure.

### 3.3. SF-ROX structures of chemically reduced *Ac*NiR   

Despite the wealth of structures of CuNiRs, there are very few structures of the reduced form of the enzyme. The best resolution structure available for a reduced copper nitrite reductase is that from *A. faecalis*, which was determined to 1.85 Å resolution some ten years ago (Wijma *et al.*, 2007 ▸). There is no XFEL structure of the reduced form of the enzyme from any species.

The structure of *Ac*NiR in the chemically reduced state (SF-ROX^RED^) obtained using 33 large colourless crystals was refined to a resolution of 1.6 Å (Table 1 ▸). The SF-ROX^RED^ T1Cu site showed a marked difference from the SF-ROX^OX^ structure, with two positions of the copper refined with occupancies of 0.7 and 0.3, respectively [Fig. 3 ▸(*b*)]. As the T2Cu site is fully reduced (as indicated by the absence of liganded water), both positions of T1Cu represent the reduced status of copper. Met141 is positioned in a single conformation away from His145, allowing a water molecule to fill the free space, making strong hydrogen bonds to both Met141 and His145. The loop (residues 187–206) undergoes a significant movement compared with that in the SF-ROX^OX^ structure (Supplementary Fig. S1).

**Figure 5:**
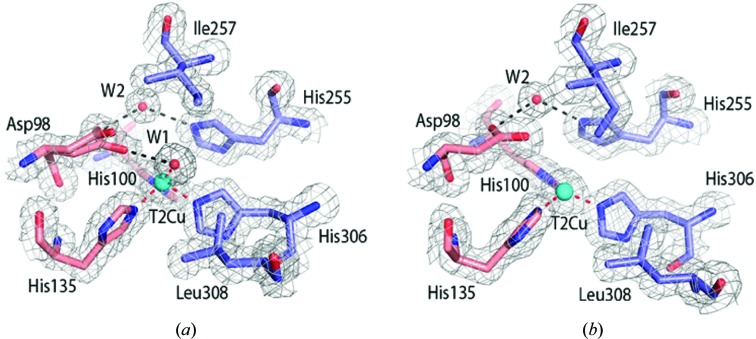
The T2Cu sites of oxidized and reduced *Ac*NiR determined by SF-ROX. (*a*) Oxidized T2Cu site with two conformations of Asp98, water W1 bound to T2Cu, and Ile257 allowing space for this water. (*b*) Reduced T2Cu site (SF-ROX^RED^). The T2Cu water ligand is lost upon reduction. Only a single Asp98 (Asp_CAT_) conformation is present. The Ile257 side chain flips down to partially fill the space vacated by the water ligand. 2*F*_o_ − *F*_c_ electron density is contoured at the 1σ level and is shown as a grey mesh. Atoms are coloured by element, with different colour schemes used for the different chains. The T2Cu is shown as a cyan sphere and water molecules are shown as small red spheres. Metal-coordinating bonds are shown as red dotted lines. Selected hydrogen bonds are shown as black dotted lines.

W1 is lost from the T2Cu site on chemical reduction, producing a tricoordinate T2Cu site with three histidine residues ligating the copper. The T2Cu also drops 0.5 Å into the histidine plane upon reduction. The electron density of the side chain of Ile257 revealed that the CD_1_ side chain flips down to partially occupy the active-site cavity space vacated by the water ligand [Fig. 5 ▸(*b*)]. The Ile257 CD_1_–T2Cu distance decreases from 5.2 to 3.6 Å, reducing the volume of and increasing the steric restraints on the active-site cavity. The distorted proximal Asp98 conformation seen in the SF-ROX^OX^ structure is not visible here, with the residue adopting the original proximal conformation. The bridging water connecting Asp_CAT_ to His_CAT_ is positioned as in the SF-ROX^OX^ structure [Fig. 5 ▸(*a*)]. The loss of water at the T2Cu in the SF-ROX^RED^ structure and the colourless nature of the crystals confirm that this structure represents the damage-free structure of the chemically reduced enzyme. In X-ray radio­lysis experiments T1Cu is reduced but T2Cu remains four-coordinate with the ligated water ligand intact (Hough *et al.*, 2008 ▸); however, movement of the the T1Cu loop (residues 187–206) is again observed (PDB entry 2vm4).

### 3.4. pH-dependence of nitrite conformation   

Given the different binding modes of nitrite in the MSOX and SF-ROX^NIT^ structures, we investigated the pH dependence of the nitrite conformation using the in-house copper-anode X-ray generator at the Barkla Laboratory equipped with an EIGER R 4M detector. The highly efficient photon-counting detector together with low-dose data collection allows complete data collection without the conversion of nitrite to NO, thus allowing the determination of NO_2_^−^-bound *Ac*NiR structures at a variety of pH values. The resolution limit of these data sets was restricted to 1.5 Å owing to the geometrical constraints of the in-house experimental setup (Supplementary Table S1). The structure at pH 5.0 was comparable to the SF-ROX^NIT^ structure, with both ‘top-hat’ and ‘side-on’ conformations of NO_2_^−^ with 0.5 occupancy each. The Asp_CAT_ residue has two conformations, with the gatekeeper conformation corresponding to the ‘top-hat’ binding mode of NO_2_^−^ [Figs. 4 ▸(*a*) and 4 ▸(*b*)]. At pH 5.5 both the NO_2_^−^ and Asp_CAT_ conformations are present in equal proportions, but several changes are noticeable in the structure. The Met141 residue protecting His145 from water binding at the short distance has a single conformation [Supplementary Fig. S3(*a*)]. At pH 5.5 Met141 adopts two conformations with half occupancy each. This allows a partial water molecule to hydrogen-bond directly to His145 and create a water network to the protein surface which ends close to the low-density loop region. At pH 6.0 the original conformation of Met141 is lost, the water hydrogen-bonded to His145 is fully occupied and the side chain of Trp144 flips 180°. A major movement occurs in the external loop [residues 192–207; Supplementary Figs. S4(*a*) and 4(*c*)], with residues 195–201 regaining almost full occupancy. The crystal structure of *Ax*NiR complexed with cytochrome *c*_551_ (PDB entry 2zon) shows the *Ax*NiR–Cyt *c*_551_ interface aligned directly on top of the equivalent loop [Supplementary Fig. S4(*d*); Nojiri *et al.*, 2009 ▸]. No changes are visible in the T2Cu geometry. Finally, at pH 6.5 few differences are observed around the T1Cu apart from both conformations of Trp144 being present. The outer loop is fully stabilized in its new conformation. The T2Cu site is changed significantly, with a single conformation of Asp_CAT_, and NO_2_^−^ is in a side-on conformation [Fig. 4 ▸(*c*)].

### 3.5. Protonation of the active-site residues in CuNiR   

**Figure 6:**
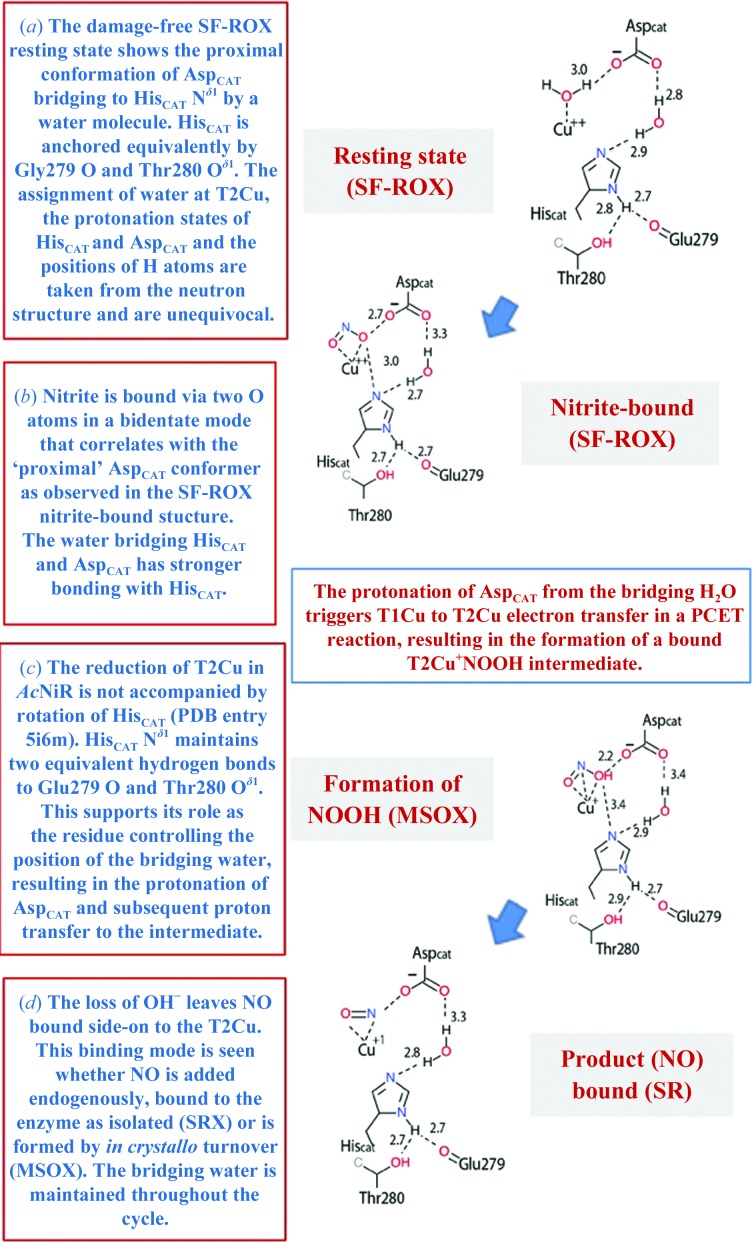
Structure-based mechanism.

The consensus view of the resting state of CuNiRs at pH values close to the optimum for activity is that Asp_CAT_ is not protonated and His_CAT_ is fully protonated, with the two residues bridged by a hydrogen-bonded water molecule (Ghosh *et al.*, 2009 ▸). In our *Ac*NiR neutron^OX^ structure the O^δ1^ and O^δ2^ atoms of Asp_CAT_ were not deuterated, as expected (Figs. 1 ▸ and 2 ▸), but, contrary to expectation, His_CAT_ lacked a deuteron at the N^∊2^ position as well. The linking water (D2) is positioned with one deuteron directed towards Asp98 O^δ1^ and one directed towards His_CAT_ N^∊2^. Moreover, the T2Cu-ligated water (D1) can clearly be modelled as a D_2_O molecule (as opposed to a D_3_O^+^ or an OD^−^ ion, which have been suggested previously as possible alternatives). The water (D2) linking His_CAT_ to O^δ1^ of Asp_CAT_ restricts the movement of the unprotonated Asp_CAT_. The bridging water is too distant to form a hydrogen bond to the O atom of the bound nitrite that interacts with Asp_CAT_. We suggest that when NO_2_^−^ binds, a proton is transferred from this water to the O^δ2^ atom of Asp_CAT_, resulting in an increase in the reduction potential to facilitate electron transfer from T1Cu to T2Cu (Fig. 6 ▸). In the complex with the reduced T2Cu, the proton is transferred from Asp_CAT_ to bound nitrite and the second proton is donated from the bridging water of His_CAT_. This residue has been shown to rotate on reduction of the T2Cu site and has a proposed role as a redox-coupled switch for proton transfer (Brenner *et al.*, 2009 ▸; Leferink *et al.*, 2011 ▸, 2012 ▸; Fukuda, Tse, Nakane *et al.*, 2016 ▸). The structure also shows that the fourth ligand of the T2Cu is D_2_O, which is consistent with proton-uptake studies, which established that two protons coupled to electron transfer are required for turnover (Brenner *et al.*, 2009 ▸). Synthetic copper complexes are able to carry out efficient NO_2_^−^ reduction with the addition of a proximal carboxylate group, analogous to Asp_CAT_, to form part of the copper(II) coordination sphere (Cioncoloni *et al.*, 2018 ▸). From a mechanistic viewpoint, our data are consistent with the binding of NO_2_^−^ to the oxidized T2Cu, resulting in displacement of the coordinated water ligand and triggering the protonation of Asp_CAT_ via the bridging water to initiate a proton-coupled electron-transfer (PCET) reaction and subsequent catalysis (Brenner *et al.*, 2009 ▸; Ghosh *et al.*, 2009 ▸).

## 4. Discussion   

A surprising feature of the previously reported damage-free XFEL structures of several CuNiRs was the absence of a water ligand to the T2Cu site. For *Af*NiR, the resting-state SFX structure had a chloride ion originating from the purification protocol ligated to T2Cu (Fukuda, Tse, Nakane *et al.*, 2016 ▸). In the case of *Gt*NiR, a sodium ion was present in the T2Cu pocket along with a low-occupancy copper (Fukuda, Tse, Suzuki *et al.*, 2016 ▸). For *Ax*NiR, the SF-ROX structure revealed an unprecedented dioxo species bound to the T2Cu site in the resting state (Halsted *et al.*, 2018 ▸), as anticipated for some time in view of a number of CuNiRs having a significant oxidase/superoxide dismutase (SOD) activity.

In contrast, our SF-ROX structure of as-isolated *Ac*NiR reported here shows that the T2Cu is ligated to a water molecule. It is the first time that this catalytically important water has been observed in a crystallographic structure obtained using femtosecond pulses from an X-ray laser. We have validated the presence of water by obtaining a 1.8 Å resolution neutron structure of a perdeuterated protein in which the water (as D_2_O) exhibits clear density for three atoms for both the catalytic and the bridging water molecules. Both damage-free structures show the *Ac*NiR T2Cu to be coordinated by three histidine residues and a single water molecule ligated in a distorted tetrahedral geometry. The distorted proximal position of Asp_CAT_ seen only in our SF-ROX structure shortens the hydrogen bond between the O^δ2^ atom of Asp98 and the T2Cu water ligand W1 from 3.5 to 3 Å.

A comparison between the SR and SF-ROX structures of NO_2_^−^-bound *Ac*NiR reveals differences at the T2Cu site. NO_2_^−^ binding to the oxidized T2Cu site has been observed in both ‘side-on’ and ‘top-hat’ modes when X-ray radiolysis is used to the drive NO_2_^−^ reduction (Horrell *et al.*, 2016 ▸). SFX structure determination of *Af*NiR revealed a single full-occupancy NO_2_^−^ molecule bound in the ‘top-hat’ position that flips to the ‘side-on’ position in SR structures (Fukuda, Tse, Nakane *et al.*, 2016 ▸). It was suggested that ‘top-hat’ to ‘side-on’ conversion occurs following the photoreduction of the T1Cu and the transfer of an electron across the Cys–His bridge, and that the ‘side-on’ conformation may represent the initial intermediate species in the catalytic reaction. This explanation is not consistent with our observations for the SF-ROX structure, in which NO_2_^−^ is present in both ‘side-on’ and ‘top-hat’ binding modes. This structure, which was obtained using single-shot XFEL pulses of pulse length <10 fs, represents a time-frozen structure in which no radiolysis can take place owing to the speed of data collection, as the X-ray pulses are shorter than even the vibrational or rotational frequencies. Both of these binding modes are also visible in low-dose data sets collected using our in-house X-ray source at a range of pH values up to pH 6.5. We therefore suggest that the generation of solvated electrons *in crystallo* by X-ray radiolysis produces a change of the pH in the active-site micro-environment of CuNiRs, shifting the geometry of Asp_CAT_ and therefore affecting the NO_2_^−^-binding mode. It has been suggested that His_CAT_ has a role as a redox-coupled switch for proton transfer (Fukuda, Tse, Nakane *et al.*, 2016 ▸), which is consistent with computational and biophysical studies showing that protonation is required for the rate-limiting intramolecular electron-transfer reaction (Ghosh *et al.*, 2009 ▸; Leferink *et al.*, 2011 ▸; Lintuluoto & Lintuluoto, 2018 ▸). Here, we observed no protonation of N^∊2^ of His_CAT_ at pD 5.4, while the linking water is neutral, suggesting that an internal change in pH is required to transfer the proton from the water (W2) to His_CAT_. The increase in pH causes a conformational change to the ‘side-on’ mode, enabling PCET-based reduction of nitrite (Fig. 6 ▸).

Even though the neutron structure was very helpful in defining the protonation states of key residues in the resting state, we note that the method has significant limitations owing to (i) lower resolution, (ii) lower completeness of data owing to Laue geometry, (iii) significantly weaker scattering lengths and cross-sections for heavier protein atoms (sulfur) and metals such as copper compared with ^2^H (Supplementary Fig. S5) and (iv) its applicability to smaller unit cells (<130 Å). XFEL-based crystallographic methods (SFX, SF-ROX, mix-and-inject SFX *etc.*) are thus currently the only methods for obtaining ‘damage-free’ structures at resolutions at which atomic details are visible with the accuracy that is necessary to define the chemistry surrounding redox centres and associated chemical reactions. Like any X-ray method, the sensitivity decreases in direct proportion to *Z* (atomic number) and hence has limitations in detecting biologically important H atoms. Combining the two approaches for the resting state has enabled us to define the protonation states of key residues experimentally for the first time.

## 5. Concluding remarks   

Structural biology continues to benefit from an expanding toolkit, the principles of which are underpinned by rigorous physics, as is demonstrated here, where unprecedented insight into the enzyme species involved in proton delivery/substrate binding in CuNiR turnover has been gained by combining results from neutron, X-ray laser, modern synchrotron and in-house laboratory X-ray sources. Neutron crystallography has remained the only radiation-damage-free macromolecular structural probe, but the advent of femtosecond crystallo­graphy with X-ray free-electron lasers provides a new opportunity in which damage-free structures can be probed using much smaller crystals and for more complex macromolecules, including membrane proteins and multi-protein complexes (Suga *et al.*, 2014 ▸, 2017 ▸; Hirata *et al.*, 2014 ▸; Nango *et al.*, 2016 ▸; Nogly *et al.*, 2018 ▸). For redox enzymes, X-ray crystallography using femtosecond X-ray lasers provides a unique opportunity to obtain damage-free structures both at cryogenic and ambient temperatures at the resolution that is needed to understand the chemistry of catalysis. The damage-free structure of the resting state of a copper nitrite reductase (CuNiR) was defined using neutron and XFEL structural data and represents the first direct comparison of neutron and XFEL structural data for any protein. The structural insights gained here will have a direct impact on computational chemistry and synthetic biology efforts for understanding proton-coupled electron-transfer events (Ghosh *et al.*, 2009 ▸) and for the design of synthetic compounds and peptides with such catalytic properties for environmental and biomedical applications (Cioncoloni *et al.*, 2018 ▸; Koebke *et al.*, 2018 ▸; Hedison *et al.*, 2019 ▸).

## Supplementary Material

PDB reference: *Achromobacter cycloclastes* CuNiR, resting state, SF-ROX structure, 6gsq

PDB reference: resting state, neutron structure, 6gtj

PDB reference: pH 5, resting state, low dose, 6gti

PDB reference: pH 5.5, resting state, low dose, 6gtk

PDB reference: pH 6, resting state, low dose, 6gtl

PDB reference: pH 6.5, resting state, low dose, 6gtn

PDB reference: nitrite-bound, SF-ROX structure, 6gt0

PDB reference: chemically reduced, SF-ROX structure, 6gt2

Supplementary Figures and Table. DOI: 10.1107/S2052252519008285/ec5013sup1.pdf

Data for neutron structure of AcNiR.: https://doi.org/10.5921/ILL-DATA.8-01-418

## Figures and Tables

**Table 1:** SF-ROX data-processing and refinement statistics Values in parentheses are for the highest resolution shell.

	SF-ROX^OX^	SF-ROX^NIT^	SF-ROX^RED^
No. of crystals	75	62	33
Images collected	1867	1257	581
Images merged	1377	1039	410
Data collection			
Space group	*P*2_1_3	*P*2_1_3	*P*2_1_3
*a* = *b* = *c* (Å)	94.95	94.92	94.61
α = β = γ (°)	90	90	90
Resolution (Å)	54.82–1.50 (1.54–1.50)	54.80–1.50 (1.54–1.50)	54.62–1.60 (1.64–1.60)
*R*_split_ † (%)	11.5 (90.4)	10.6 (85.3)	15.8 (70.8)
〈*I*/σ(*I*)〉	6.3 (2.0)	6.6 (2.3)	5.4 (2.7)
CC_1/2_ ‡	0.980 (0.157)	0.984 (0.288)	0.957 (0.384)
Completeness (%)	100.0 (100.0)	100.0 (100.0)	100.0 (100.0)
Multiplicity	220.9 (77.2)	154.7 (50.8)	66.1 (44.3)
Wilson *B* factor (Å^2^)	14.9	14.5	17.5
Refinement			
No. of unique reflections	45883 (2276)	45846 (2275)	37489 (1858)
*R*_work_/*R*_free_ (%)	14.4/17.7	14.3/17.1	16.7/19.9
No. of atoms			
Protein	2608	2595	2580
Ligand/ion	37	34	59
Water	425	418	279
*B* factors (Å^2^)			
Protein	18.9	18.7	21.9
Cu	16.5	16.2	19.5
SO_4_^2−^	33.2	41.5	
NO_2_^−^		18.5	
Malonate		37.2	30.1
Water	30.0	30.0	32.7
R.m.s. deviations			
Bond lengths (Å)	0.013	0.013	0.015
Bond angles (°)	1.612	1.594	1.534
PDB code	6gsq	6gt0	6gt2

† *R*_split_ is as defined by White *et al.* (2013 ▸).

‡ The correlation coefficient between half data sets is as defined by Karplus & Diederichs (2015 ▸).

**Table 2:** Neutron data-processing and refinement statistics for neutron^OX^ Values in parentheses are for the highest resolution shell.

Data collection	
Wavelength range (Å)	3.05–4.00
No. of images	20
Setting spacing (°)	7
Average exposure time (h)	18
Space group	*P*2_1_3
*a* = *b* = *c* (Å)	97.98
α = β = γ (°)	90
Resolution (Å)	40–1.80 (1.90–1.80)
*R*_p.i.m._ (%)	6.3 (12.7)
〈*I*/σ(*I*)〉	7.9 (3.7)
Completeness (%)	85.5 (69.8)
Multiplicity	6.5 (2.9)
Refinement	
No. of unique reflections	24728
*R*_work_/*R*_free_ (%)	23.17/27.64
No. of atoms	
Total	5659
Protein	5109
Cu	2
D_2_O	182 D_2_O [546 atoms]
O	2
*B* factors (Å^2^)	
Protein	15.2
Cu	8.6
Water	20.2
R.m.s. deviations	
Bond lengths (Å)	0.004
Bond angles (°)	0.884
PDB code	6gtj
