# Supplementary material for: Catalytically important damage-free structures of a copper nitrite reductase obtained by femtosecond X-ray laser and room-temperature neutron crystallography
Source: IUCrJ. 2019 Jun 23;6(Pt 4):761–72. doi: 10.1107/S2052252519008285 (PMC6608623; doi:10.1107/S2052252519008285)
Supplement: Supplementary file 1 [file m-06-00761-sup1.pdf]

# IUCrJ

**Volume 6 (2019)**


**Supporting information for article:**

## Catalytically important damage-free structures of a copper nitrite reductase obtained by femtosecond X-ray laser and room-temperature neutron crystallography

**Thomas P. Halsted, Keitaro Yamashita, Chai C. Gopalasingam, Rajesh T. Shenoy, Kunio Hirata, Hideo Ago, Go Ueno, Mathew P. Blakeley, Robert R. Eady, Svetlana V. Antonyuk, Masaki Yamamoto and S. Samar Hasnain**

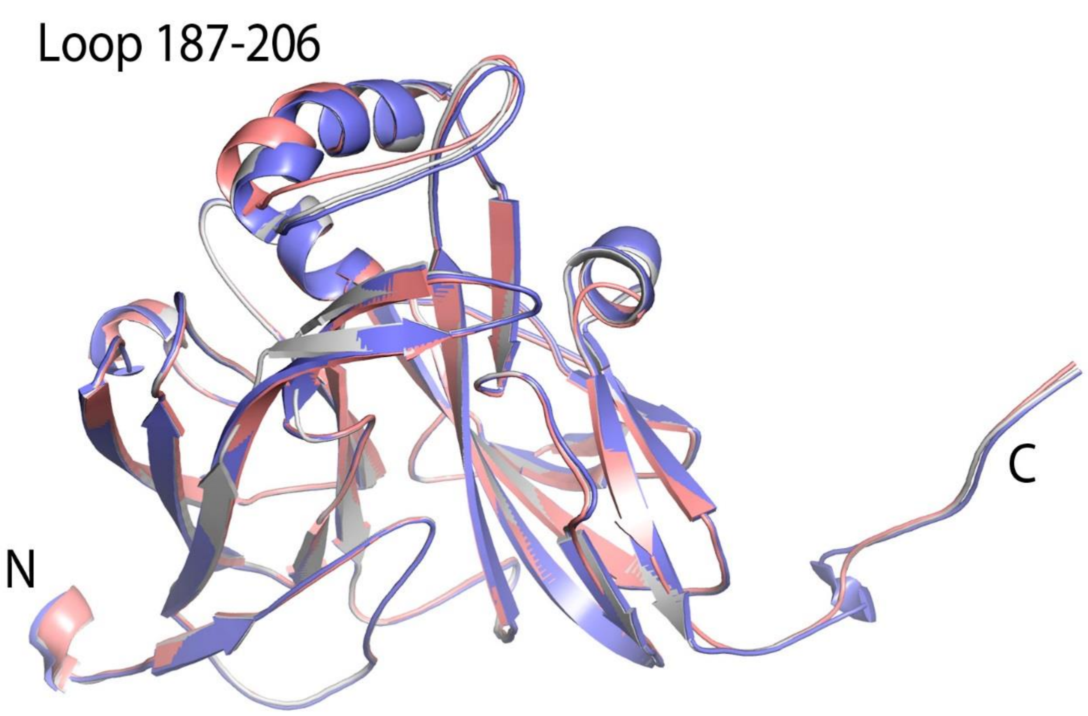


**Figure S1** Alignment of the SR-ROX$^{OX}$, SF-ROX$^{RED}$ and neutron$^{OX}$ structures. The major difference between the structures is in the surface loop region (residues 187 – 206). The loop is partially disordered in the SF-ROX$^{OX}$ structure with one conformation traceable in the electron density. The loop in the SF-ROX$^{RED}$ and neutron$^{OX}$ structures is ordered and adopts a single conformation. The surface loop is associated with the binding of redox carrier proteins such as azurin and c-type cytochromes(Nojiri *et al.*, 2009), see Figure S3d. The SF-ROX$^{OX}$ structure is shown in salmon, the SF-ROX$^{RED}$ structure in grey and the neutron$^{OX}$ structure shown in lilac.

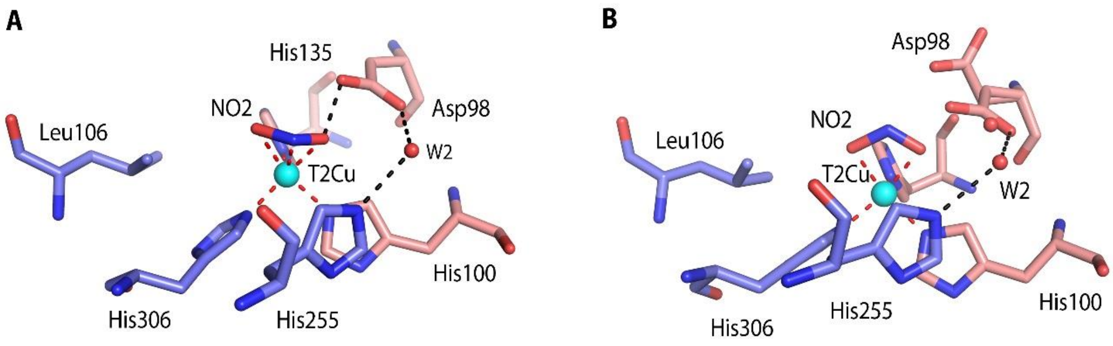


**Figure S2** Detailed view for side-on and top-hat $NO_2$—binding to T2Cu site of *Ac*NiR. **(A)** Side-on $NO_2^-$ is bound to the T2Cu at pH 6.5 with Asp98 ($Asp_{CAT}$) in proximal position. All nitrite atoms are coordinated by Cu. **(B)** Top-hat conformation of nitrite at pH 5.0 obtained using low-dose home source corresponding to gate keeper Asp98 position; The T2Cu is shown as a cyan sphere and water molecules as small red spheres. Metal-coordinating bonds are shown as red dotted lines. Selected hydrogen bonds are shown as black dotted lines.

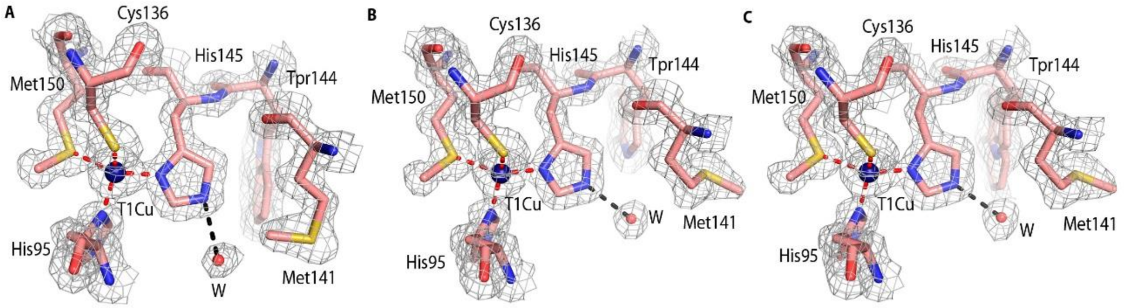


**Figure S3** pH dependent changes around the T1Cu site in $NO_2^-$-bound *Ac*NiR. **(A)** In nitrite bound structure obtained using low-dose home source, at pH 5.0 Met141 is preventing strong bonding of the water to His145. **(B)** As pH increases to 6.0, the Met141 residue shifts away and allows a water molecule to bind to T1Cu His145 ligand at 2.7 Å distance. The Tpr144 side-chain then rotates 180° underneath the surface loop. **(C)** At pH 6.5 His145 is still bound to water ligand at the short distance as in C but Tpr144 has two conformations. Atoms are coloured by element. 2Fo-Fc electron density is contoured at the 1σ level and shown as a grey mesh. Atoms are coloured by element. T1Cu is shown as dark blue sphere, water as small red sphere.

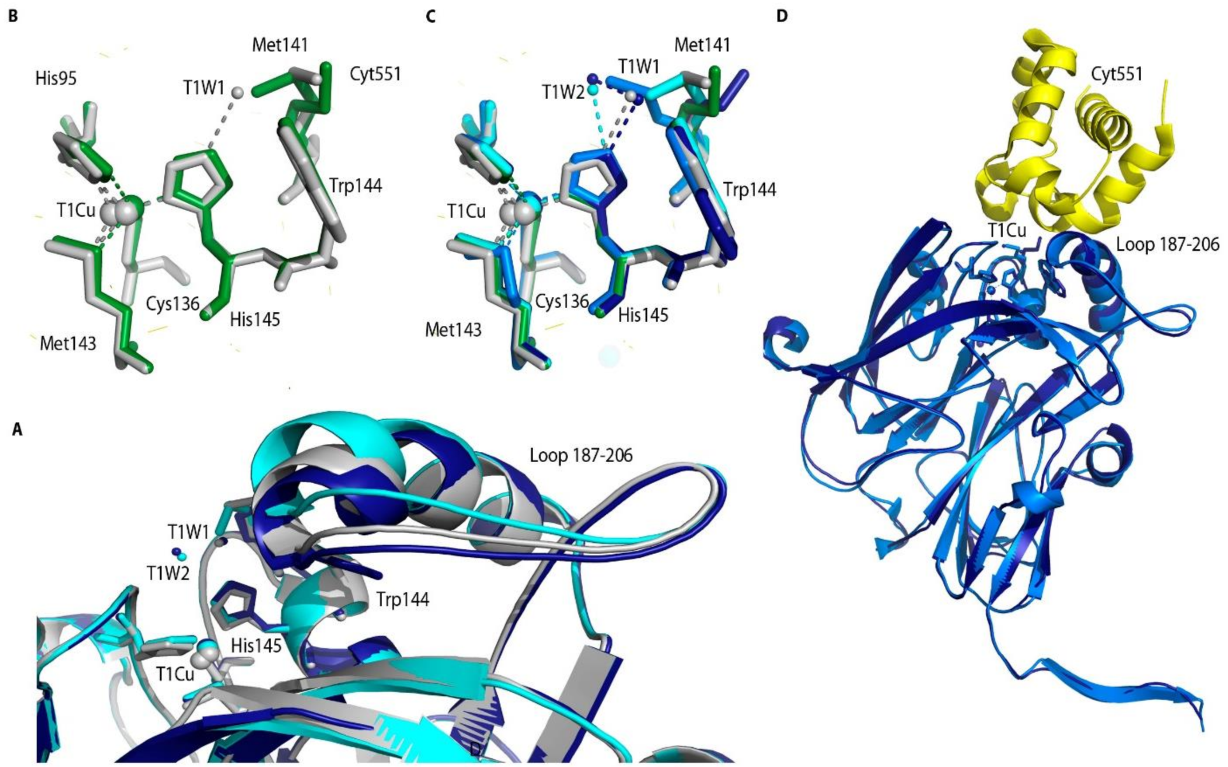


**Figure S4** Comparison of T1Cu and the interacting loop in *Ax*NiR:Cyt$C_{551}$ complex with *Ac*NiR structures. **(A).** Superposition of the SF-ROX$^{RED}$ (grey) with $NO_2^-$-bound *Ac*NiR structures at pH 6.5 (dark blue) and 5.0 (cyan). At both pH 5.0 and pH 5.5, the loop (187-206) is disordered with limited electron density along the chain. At pH 6.0 and 6.5, loop becomes ordered with a single clear conformation similar to SF-ROX$^{RED}$ structure. **(B)** Superposition of T1Cu site residues for SF-ROX$^{RED}$ (grey) and SF-ROX$^{OX}$ (green) clearly showing different conformation of the Cu ligands and different positions of Cu. Both positions of T1Cu in SF-ROX$^{RED}$ are distinct from the position in SF-ROX$^{OX}$. **(C)** Superposition of T1Cu site residues for *Ac*NiR of SF-ROX$^{RED}$ (grey), SR-ROX$^{OX}$(green), $NO_2^-$-bound at pH 6.5 (dark blue) and 5.0 (cyan) and *Ax*NiR:Cyt$C_{551}$ (blue) showing correlated positions of Met141 and T1Cu-W1/W2. **(D)** *Ax*NiR from cytochrome $c_{551}$ – *Ax*NiR complex (PDB: 2ZON) has similar conformation of the flexible surface loop (187-206) as $NO_2^-$-bound *Ac*NiR at pH 6.5 and reduced *Ac*NiR. *Ax*NiR is shown in blue, $NO_2^-$-bound *Ac*NiR in dark blue and cytochrome $c_{551}$ in the complex in yellow (reduced *Ac*NiR not shown for clarity).

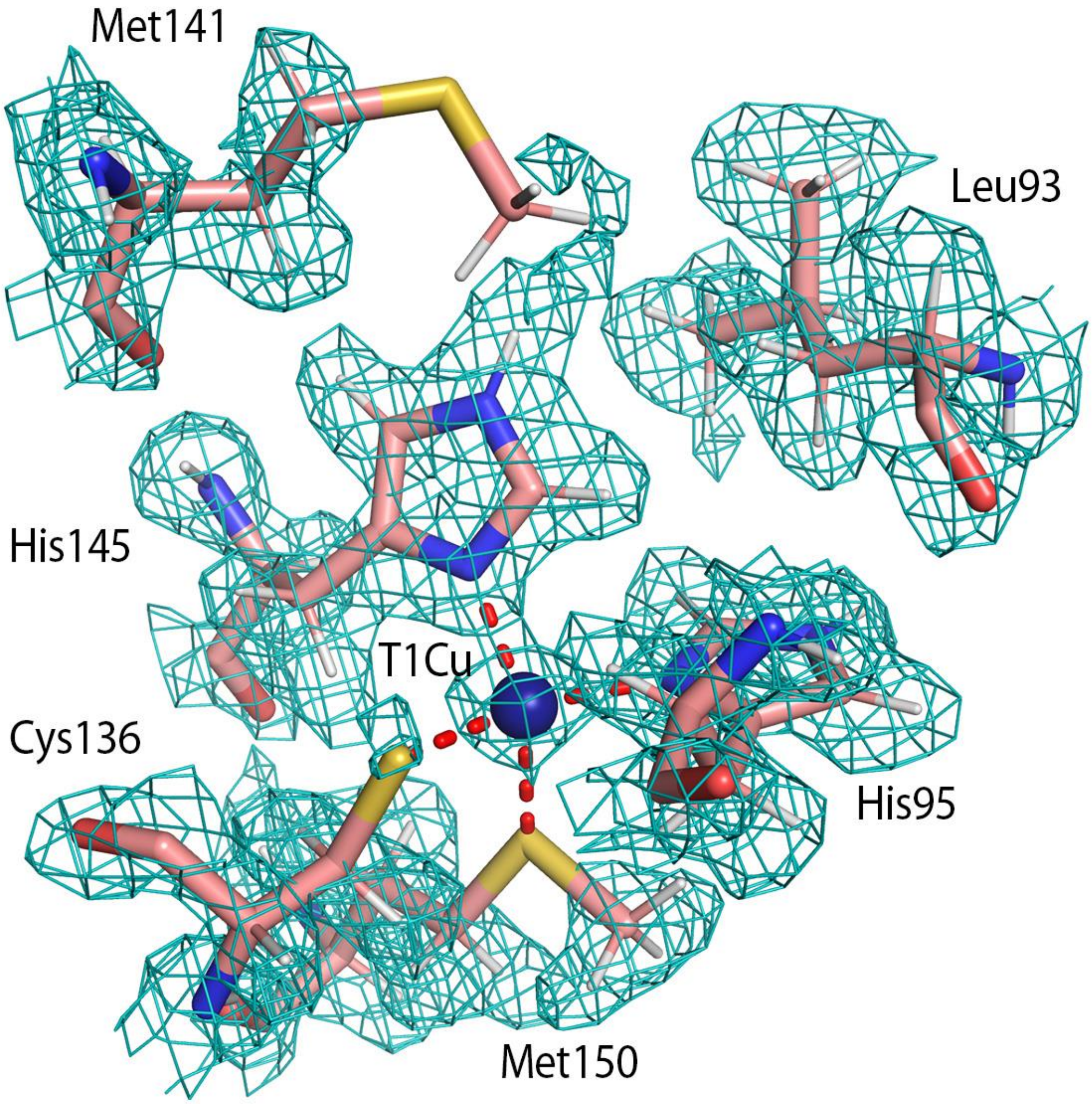


**Figure S5** T1Cu site determined by neutron crystallography. The protonation states of the T1Cu site residues are clearly seen. 2Fo-Fc nuclear scattering map is contoured at the 1σ level and shown as cyan mesh. Atoms are coloured by element. The T1Cu is shown as a cyan sphere. Metal-coordinating bonds are shown as red dotted lines. Selected hydrogen bonds are shown as black dotted lines. Nuclear density is missing on the sulphur atoms of Met141 and Met150. The density for Cu is similar to the density of hydrogen atoms while the density of Cys136 sulphur atom is fairly weak.

**Table S1** In-house X-ray source data processing and refinement statistics

The values in parentheses correspond to the highest resolution bin.

| | | pH 5.0 | pH 5.5 | pH 6.0 | pH 6.5 |
|---|---|---|---|---|---|
| **Data collection** | | | | | |
| Space group | | $P2_13$ | $P2_13$ | $P2_13$ | $P2_13$ |
| Unit cell dimensions | | | | | |
| a=b=c (Å) | | 95.42 | 95.47 | 95.63 | 95.53 |
| α=β=γ (°) | | 90 | 90 | 90 | 90 |
| Resolution (Å) | | 7.90 – 1.50 (1.53 – 1.50) | 8.19 – 1.50 (1.53 – 1.50) | 8.84 – 1.50 (1.53 – 1.50) | 7.90 – 1.50 (1.53 -1.50) |
| $R_{p.i.m.}$ (%) | | 3.6 (47.3) | 3.6 (50.6) | 2.6 (20.1) | 2.5 (24.1) |
| $\langle I/\sigma(I) \rangle$ | | 14.7 (1.4) | 14.1 (1.3) | 16.6 (3.1) | 17.8 (2.5) |
| $CC_{1/2}$* | | 0.999 (0.653) | 0.999 (0.657) | 0.999 (0.917) | 0.999 (0.876) |
| Completeness (%) | | 99.7 (98.3) | 99.7 (96.4) | 99.7 (98.5) | 99.6 (95.3) |
| Redundancy | | 6.2 (3.4) | 6.2 (3.4) | 6.1 (3.3) | 6.1 (3.4) |
| Wilson B-factor ($Å^2$) | | 11.3 | 11.7 | 9.4 | 10.7 |
| **Refinement** | | | | | |
| No. of unique reflections | | 46390 (2247) | 46462 (2204) | 46648 (2249) | 46466 (2186) |
| $R_{work}/R_{free}$ (%) | | 15.3/17.8 | 16.0/18.5 | 14.7/16.8 | 15.2/18.1 |
| No. atoms | | | | | |
| Protein | | 2630 | 2650 | 2641 | 2643 |
| Ligand/ion | | 29 | 22 | 22 | 19 |
| Water | | 468 | 487 | 504 | 498 |
| B-factors ($Å^2$) | | | | | |
| Protein | | 14.7 | 15.0 | 12.9 | 14.1 |
| Cu | | 10.9 | 11.6 | 9.8 | 11.5 |
| $NO_2^-$ | | 12.3 | 12.9 | 12.4 | 15.5 |
| Malonate | | 25.3 | 25.6 | 24.4 | 28.4 |
| Water | | 27.2 | 27.5 | 25.7 | 26.5 |
| R.m.s deviations | | | | | |
| Bond length (Å) | | 0.013 | 0.013 | 0.014 | 0.014 |
| Bond angles (°) | | 1.668 | 1.649 | 1.789 | 1.765 |
| PDB access code | | 6GTI | 6GTK | 6GTL | 6GTN |

*The correlation coefficient between half datasets is defined by here (Karplus & Diederichs, 2015).
